# The role of the Ge mole fraction in improving the performance of a nanoscale junctionless tunneling FET: concept and scaling capability

**DOI:** 10.3762/bjnano.9.177

**Published:** 2018-06-22

**Authors:** Hichem Ferhati, Fayçal Djeffal, Toufik Bentrcia

**Affiliations:** 1 LEA, Department of Electronics, University Mostefa Benboulaid-Batna 2, Batna 05000, Algeria; 2 LEPCM, University of Batna 1, Batna 05000, Algeria

**Keywords:** ambipolar conduction, heterojunctions, junctionless tunneling field-effect transistor (JL TFET), nanoscale, SiGe

## Abstract

In this paper, a new nanoscale double-gate junctionless tunneling field-effect transistor (DG-JL TFET) based on a Si_1−_*_x_*Ge*_x_*/Si/Ge heterojunction (HJ) structure is proposed to achieve an improved electrical performance. The effect of introducing the Si_1−_*_x_*Ge*_x_* material at the source side on improving the subthreshold behavior of the DG-JL TFET and on suppressing ambipolar conduction is investigated. Moreover, the impact of the Ge mole fraction in the proposed Si_1−_*_x_*Ge*_x_* source region on the electrical figures of merit (*FoMs*) of the transistor, including the swing factor and the *I*_ON_/*I*_OFF_ ratio is analyzed. It is found that the optimized design with 60 atom % of Ge offers improved switching behavior and enhanced derived current capability at the nanoscale level, with a swing factor of 42 mV/dec and an *I*_ON_/*I*_OFF_ ratio of 115 dB. Further, the scaling capability of the proposed Si_1−_*_x_*Ge*_x_*/Si/Ge DG-HJ-JL TFET structure is investigated and compared to that of a conventional Ge-DG-JL TFET design, where the optimized design exhibits an improved switching behavior at the nanoscale level. These results make the optimized device suitable for designing digital circuit for high-performance nanoelectronic applications.

## Introduction

In the last years, the continuous miniaturization of nanoscale transistors induces new challenges including short-channel effects (SCEs) and high power consumption, which prevent incorporating conventional metal-oxide semiconductor field-effect transistors (MOSFETs) and their complements in nanoelectronic circuit designs [1–4]. In this context, small swing-switch devices such as double-gate tunneling field-effect transistors (DG TFETs) are gaining attention because of their good subthreshold characteristics, high scalability and low OFF-current [5–8]. The main idea behind this innovative device is the use of a gated p-i-n diode, the working mechanism of which is based on a quantum band-to-band tunneling effect. This makes it more immune against the undesired SCEs and enables a better scaling capability. Despite such attractive properties, DG TFETs still suffer from other issues mainly related to the relatively low ON-state current and the severe ambipolar conduction, which make it extremely challenging for designing high-performance digital nanocircuits [7–9]. Consequently, intensive efforts have been paid to address these limitations by proposing new designs based on heterostructures, gate engineering and high-*k* dielectrics [9–12]. Nanoscale DG TFETs are believed to face an upward amendment to meet the difficulty of decreasing the huge thermal budget required for the formation of the gated p-i-n diode structure. Moreover, in spite of the actual mature experimental techniques, realizing metallurgical junctions in sub-32 nm nodes is considered extremely difficult [13–15]. For this purpose, the junctionless (JL) design is considered the best approach to avoid the above outlined experimental limitations and achieve significant improvements regarding the transistor manufacturing cost [14–17]. The JL technology is considered to be cost-effective and allows avoiding the high thermal budget [15–17]. The concept of a gated source is used for the JL technology in order to ensure the band-to-band tunneling effect, while materials with high work function are required to generate the tunnel current. In other words, the channel is assumed to be a uniformly and highly doped *n*-type material, and in order to ensure the band-to-band tunneling effect, the source is supported with a control gate to make the device behave like a conventional p-i-n-based TFET [18]. However, the most pronounced drawbacks associated with the DG TFETs design also persist in the JL structure, namely the low ON-state current, the high leakage current and parasitic ambipolar conduction, which can eventually prevent the application in high-performance nanoelectronic circuits. The DG-JL TFET design can pave the way to reduce the fabrication cost, but it exhibits degraded electrical FoMs. Several recently published works are focused on improving the multi-gate JL TFET by suggesting design improvements such as gate underlap and overlap, introducing III–V materials and source/drain engineering [19–24]. Additional approaches are in fact required in order to push the limits of the DG-JL TFET performance and achieve energy-efficient and scalable transistors. To the best of our knowledge, no design approach based on Si_1−_*_x_*Ge*_x_*/Si/Ge heterochannels with optimized Ge content was proposed to improve the electrical performance and to suppress the parasitic ambipolar conduction in DG-JL TFETs. We present in this paper, a new DG-HJ-JL TFET design to achieve improved electrical FoMs and reduced fabrication cost. An exhaustive numerical study of the electrical behavior of the proposed device at the nanoscale level is performed using the ATLAS 2-D simulation software [25]. Further, the impact of the Ge content on the electrical performance of the transistor is investigated. It is found that the proposed design offers between improved derived current capability and reduced ambipolar conduction compared to a conventional DG Ge-JL-TFET design. In order to consolidate our investigation, the scaling capability of the proposed design is investigated and compared to that of the conventional counterpart, where the proposed structure demonstrates a superior switching behavior. This makes the optimized structure a potential alternative for providing energy-efficient transistors with suppressed ambipolar conduction for designing high-performance nanoelectronic circuits at low manufacturing cost.

## Numerical Simulations

**Figure 1:**
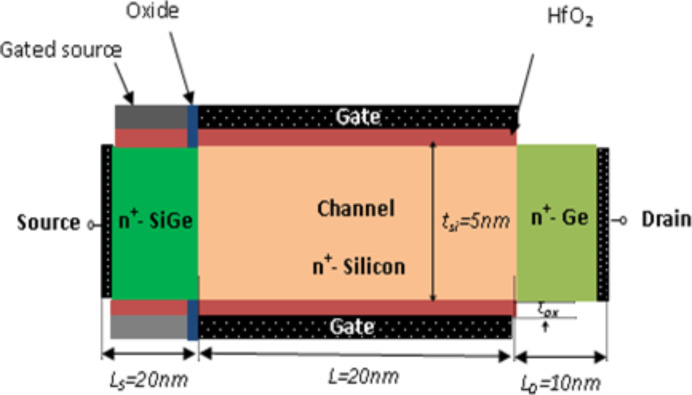
Schematic of the investigated DG-HJ-JL TFET with *N*_d_ = 1·10^19^ cm^−3^ and *t*_ox_ = 3 nm.

Figure 1 describes schematically the investigated DG-HJ-JL TFET structure. The cornerstone of the proposed design is the assumption of a uniformly and highly doped heterochannel (Si_1−_*_x_*Ge*_x_*/Si/Ge), which can be indicated by *n*^+^/*n*^+^/*n*^+^. In addition, the proposed design is suggested with a HfO_2_ gate dielectric in order to ensure a good electrostatic control of the channel, with *t*_ox_ representing its thickness. Moreover, the material of the gated source is assumed to have a high work function value of 5.6 eV in order to guarantee the tunnel effect, while the work function of the channel gate is set equal to 4.3 eV. In Figure 1, *L* is the channel length, *t*_ch_ refers to the channel thickness, *N*_d_ is the doping concentration of the channel, and *L*_s_ and *L*_d_ denote the extension lengths of source and drain, respectively.

The accurate modeling of the nanoscale DG-HJ-JL TFET requires taking into account quantum-confinement effects, which lead to some modeling bottlenecks associated with the models of the carrier density gradient. Furthermore, since the investigated transistor is considered as a quantum mechanical device, complicated systems of equations resulted from the necessity of considering the band-to-band quantum tunneling effects. These nonlinear equations impose many mathematical difficulties, which complicate the analytical modeling of the nanoscale device performance. Numerical approaches are used to deal with the above outlined problems. The ATLAS 2D device simulator using the S-PISCES module has emerged recently as a useful and realistic tool for numerically modeling the electrostatic behavior of transistors [25].

The electrostatic behavior of the investigated nanoscale (Si_1-x_Ge_x_/Si/Ge) DG-HJ-JL TFET including the tunnel effects is modeled using the nonlocal-BTBT command, which takes into account nonlocal band-to-band quantum tunneling [25]. In this perspective, the tunnel current is generated near the source/channel junction and can be characterized by a transfer of electrons and holes across this junction. Hence, the tunneling current for an electron with longitudinal energy *E* and transverse energy *E*_T_ can be expressed as follows [25]:

[1]
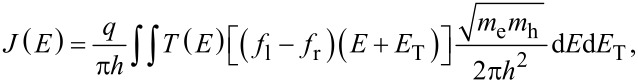


where *T*(E) represents the tunneling probability of the electrons, *q* is the electron charge, *m*_e_ and *m*_h_ are the effective masses of electrons and holes, respectively, *h* is the Planck constant. *f*_l_ and *f*_r_ are the Fermi–Dirac distributions on the left and the right side of the source/channel junction, respectively:

[2]
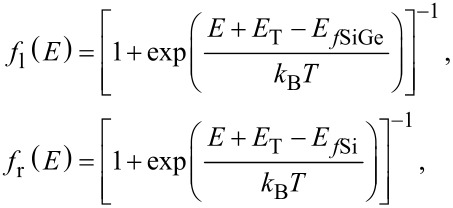


where, *E_f_*_SiGe_ and *E_f_*_Si_ are the Fermi levels at the Si_1−_*_x_*Ge*_x_* source and Si channel regions, respectively, *k*_B_ is the Boltzmann constant and *T* the temperature.

In order to reflect accurately the device behavior for very short dimensions like in our case, the modified drift–diffusion model, which includes other effects related to the short-channel nature of the investigated transistor and to quantum effects is used. Further, the gradient density model is also included, which consists of the quantum correction associated with the local potential to the carrier temperatures in the current equations [25]. Moreover, models for carrier recombination (Shockley–Read–Hall (SRH), Auger and surface recombination) are also adopted [26]. In fact, the carrier mobility mainly depends on three quantities, transverse and parallel electric field, doping and temperature, which were combined using Matthiessen’s formula. Accordingly, the Lombardi model (CVT) is used to express the carrier mobility in the channel [27]. Moreover, the intrinsic parameters of the materials (Si, Si_1−_*_x_*Ge*_x_* and Ge) such as band gap, mobility and the density of states were considered to be dependent on the Ge mole fraction (*x*_Ge_). It should be noted that the *Ge* mole fraction is varied from 0 to 0.7. This corresponds to the experimental limit for growing Si_1−_*_x_*Ge*_x_* when avoiding interfacial defects at the considered device thickness *t*_ch_ = 5 nm [28].

## Results and Discussion

**Figure 2:**
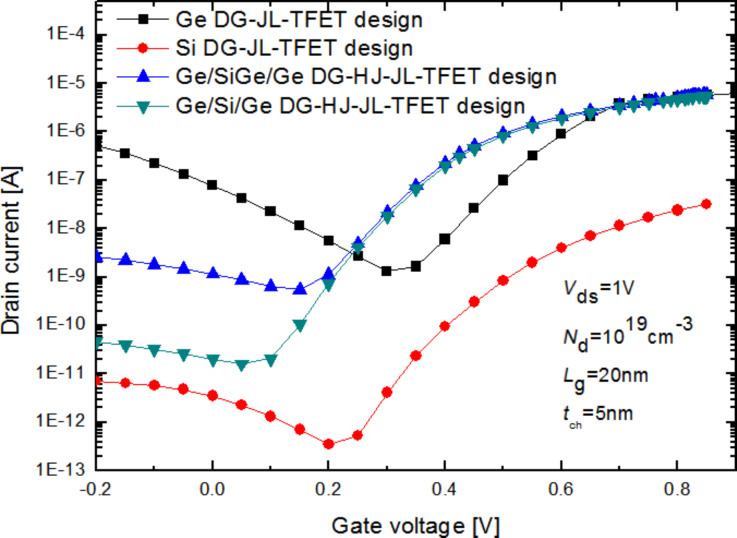
Drain current as a function of the applied gate voltage for the DG-HJ-JL TFET proposed with different heterochannel configurations compared to that of the conventional homochannel designs (*L*_g_ = 20 nm, *L*_s_ = 10 nm and *N*_d_ = 1·10^19^ cm^−3^, *t*_ch_ = 5 nm and *V*_ds_ = 1 V).

The main idea behind the proposed design is a modified heterostructured channel. In this context, it seems important to analyze the electrical behavior of the proposed design with considering different material configurations at the source, drain and channel regions in order to distinguish which heterochannel design provides the best electrical performance. Figure 2 depicts *I*_ds_–*V*_gs_ characteristics of the proposed design with different material configurations of the heterochannel compared to that of the conventional designs with *L*_g_ = 20 nm, *L*_s_ = 10 nm and *N*_d_ = 1·10^19^ cm^−3^.

The proposed DG-HJ-JL TFET design with Ge/Si/Ge channel structure exhibits better electrostatic behavior and less parasitic ambipolar conduction than the other designs. In fact, this behavior can be attributed to two essential effects: Firstly, the enhanced tunneling current resulting from the low tunneling barrier giving rise to a higher probability of electron transfer at the source/channel interface. Secondly, the heterostructure at the channel/drain interface can be beneficial for sufficiently enlarging the tunneling barrier under reverse-bias conditions in order to effectively suppress the undesired ambipolar conduction. Moreover, we can notice that the conventional design with Si channel shows a reduced OFF-state current compared to that of the investigated heterochannel designs, which is mainly due to the high band-gap energy and the low electron mobility associated to Si. On the other hand, the conventional Ge-DG-JL TFET design provides higher ON-state current. This is due to the smaller band gap energy of Ge, yielding a higher tunneling efficiency. Moreover, the higher electron mobility of Ge (3900 cm^2^·V^−1^·s^−1^) contributes to the increased drain current at the threshold voltage as compared to the conventional design with silicon (1400 cm^2^·V^−1^·s^−1^) channel. A suitable choice of the channel material can offer the possibility of enhancing the *I*_ON_/*I*_OFF_ ratio as well as achieving a lower swing factor. For this purpose, introducing Si_1−_*_x_*Ge*_x_* material at the source side can be useful for achieving an improved electrical behavior through modulating the tunneling barrier width at the source/channel junction by varying the Ge concentration.

**Figure 3:**
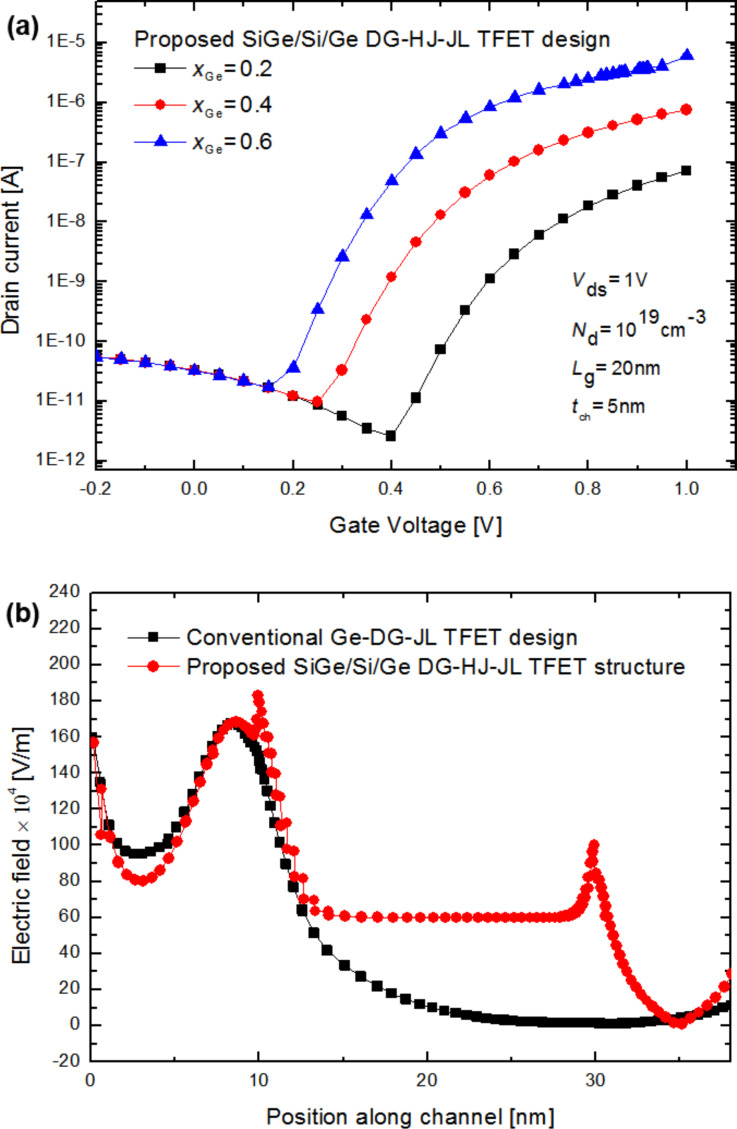
(a) Transfer characteristics of the proposed Si_1−_*_x_*Ge*_x_*/Si/Ge DG-HJ-JL TFET as a function of the Ge concentration with *N*_d_ = 1·10^19^ cm^−3^, *V*_ds_ = 1 V, *t*_ox_ = 2 nm, *L*_g_ = 20 nm and *t*_ch_ = 5 nm. (b) Distribution of the electric field along the channel of the proposed Si_1−_*_x_*Ge*_x_*/Si/Ge DG-HJ-JL TFET structure and the conventional Ge-DG-JL TFET design with *N*_d_ = 1·10^19^ cm^−3^, *t*_ch_ = 5 nm, *t*_ox_ = 2 nm, *L*_g_ = 20 nm and *L*_s_ = 10 nm.

Figure 3a shows the transfer characteristics associated of the proposed Si_1−_*_x_*Ge*_x_*/Si/Ge DG-HJ-JL TFET design with different Ge mole fractions. Increasing the Ge content leads to an increase of the drain current. This is mainly due to the enhanced carrier mobility caused by the increased Ge content. Moreover, introducing SiGe at the source side can be effective for reducing the tunneling barrier. Besides, the Ge concentration increase induces a lowering of the tunneling barrier, which enables enhancing the derived current capability as shown in Figure 3a. It can be also concluded that the Ge mole fraction modulates the threshold voltage for the tunnel-current generation, which can in turn influence greatly the subthreshold behavior of the device. Moreover, the proposed Si_1−_*_x_*Ge*_x_*/Si/Ge heterochannel enables a superior control of the channel conductivity through modulating the electric field at the heterojunction interfaces. In this regard, it is of great importance to illustrate the electric field distribution for a better understanding of the physical rules governing the obtained improvements of the electrostatic behavior. Figure 3b compares the distribution of the electric field along the channel of the proposed Si_1−_*_x_*Ge*_x_*/Si/Ge DG-HJ-JL TFET design to that of the conventional Ge-DG-JL TFET counterpart. Clearly, a considerable change in the electric field distribution can be achieved by including the heterochannel, with higher electric field arising in the source/channel interface as well as along the channel. This indicates that by a proper choice of the channel material, we can achieve an enhanced electrostatic behavior. This enables improving the carrier transport efficiency and thereby the device derived current capability at the nanoscale level.

**Figure 4:**
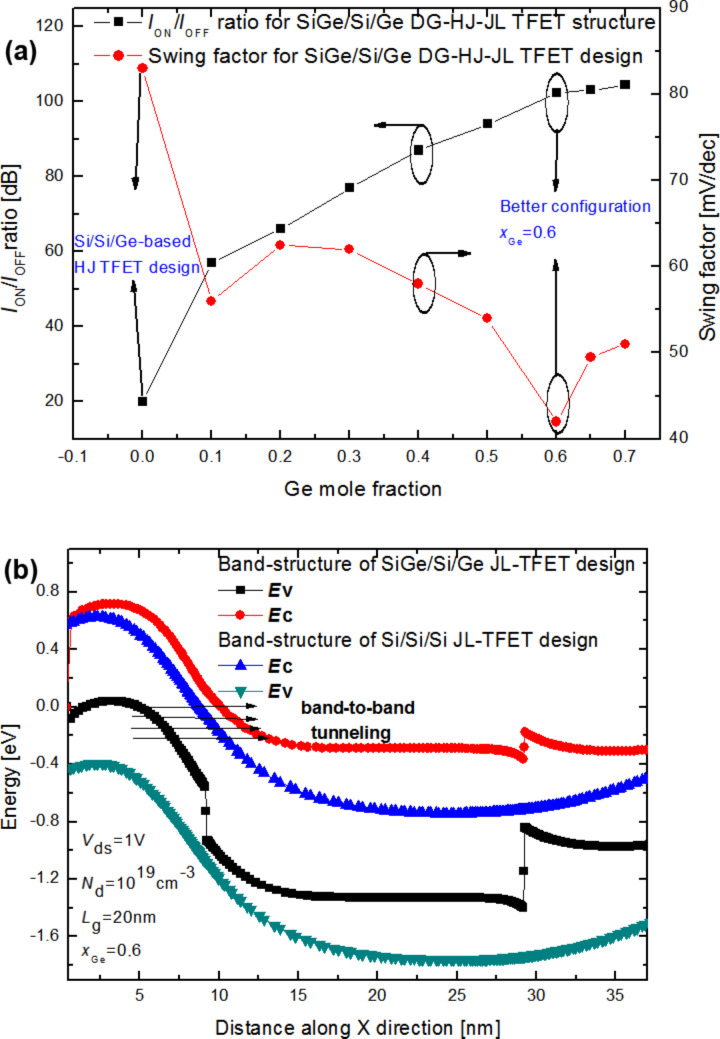
(a) Subthreshold swing factor and the *I*_ON_/*I*_OFF_ ratio as functions of the Ge mole fraction for the investigated Si_1−_*_x_*Ge*_x_*/Si/Ge DG-HJ-JL TFET structure with *N*_d_ = 1·10^19^ cm^−3^, *V*_ds_ = 1 V, *t*_ox_ = 2 nm, *L*_g_ = 20 nm and *t*_ch_ = 5 nm. (b) Comparison of the band diagrams of the proposed Si_1−_*_x_*Ge*_x_*/Si/Ge DG-HJ-JL TFET design and the conventional structure with uniform Si channel*.*

In order to get a qualitative idea about the impact of the Ge concentration on the electrical performance of the proposed DG-HJ-JL TFET design, Figure 4a shows both *I*_ON_/*I*_OFF_ ratio and the subthreshold swing factor as functions of the Ge mole fraction. By increasing the Ge content, the *I*_ON_/*I*_OFF_ ratio increases significantly to reach its maximum for a Ge mole fraction value of 0.6 and saturates after this value. Moreover, the *I*_ON_/*I*_OFF_ ratio of the proposed DG-HJ-JL TFET is higher than that of the conventional structure with uniform channel. This can be attributed to the enhanced tunneling current resulting from the change of the tunneling barrier with increasing Ge content. Figure 4b compares the band diagrams of the DG-HJ-JL TFET design and the conventional structure with uniform Si channel. Figure 4b reveals that by introducing Si_1−_*_x_*Ge*_x_*, the tunneling barrier height at the source–channel junction decreases and a higher tunneling current can be generated when the band alignment at the junction is satisfied.

In addition, Figure 4 shows the complex subthreshold behavior. The Ge mole fraction induces a highly non-linear behavior of the swing factor as it is shown in Figure 4a. This phenomenon can be ascribed to the quantum nature of the band-to-band tunneling effects, and determining the Ge concentration that provides an enhanced subthreshold behavior seems to be very complex at the nanoscale level. The swing factor decreases significantly above *x*_Ge_ = 0.3, which can be explained by the effect of the tunneling barrier height on the device subthreshold behavior. At a Ge mole fraction of 0.6, a good trade-off between derived current capability and subthreshold behavior is obtained, with an *I*_ON_/*I*_OFF_ ratio value of 115 dB and a swing factor value of 42 mV/dec at the nanoscale level (*L*_g_ = 20nm) as it is illustrated in Figure 4a.

**Figure 5:**
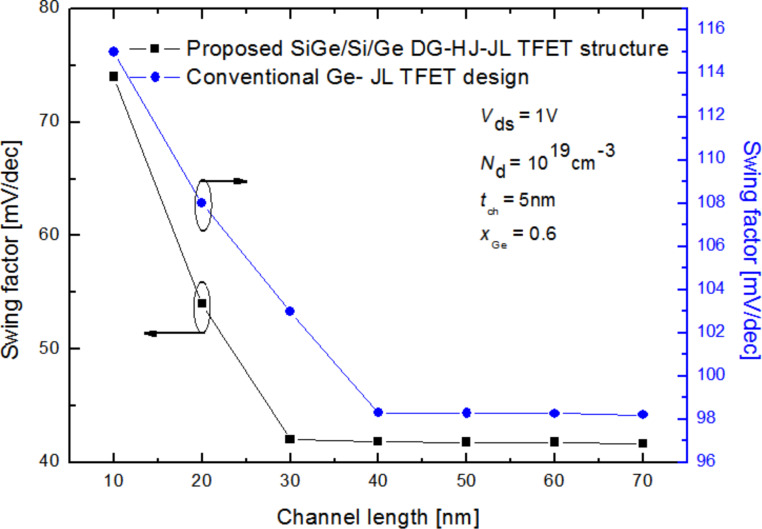
Subthreshold swing factor as a function of the channel length for both the proposed Si_1−_*_x_*Ge*_x_*/Si/Ge DG-HJ-JL TFET and the conventional Ge-DG-JL TFET with *N*_d_ = 1·10^19^ cm^−3^, *V*_ds_ = 1 V, *t*_ox_ = 2 nm, *L*_s_ = 10 nm and *t*_ch_ = 5nm.

More importantly, we analyze the impact of the optimized Si_1−_*_x_*Ge*_x_*/Si/Ge heterochannel structure on the device scaling capability for high-performance nanoelectronic applications. Figure 5 depicts the swing factor as a function of the transistor channel length for both the proposed Si_1−_*_x_*Ge*_x_*/Si/Ge DG-HJ-JL TFET design and the conventional Ge-DG-JL TFET structure with *t*_ch_ = 5 nm, *x*_Ge_ = 0.6 and *N*_d_ = 1·10^19^ cm^−3^. The optimized design provides better scaling capability than the conventional one and exhibits a faster decrease of the swing factor as a function of the channel length. This behavior can be explained by the improved electrostatic response offered by the heterochannel.

For the completeness of this work, we analyze the improvements of the proposed design compared to conventional TFET devices with regard to the electrical performance. Table 1 summarizes an overall comparison of electrical metrics between the proposed Si_1−_*_x_*Ge*_x_*/Si/Ge DG-HJ-JL TFET, the conventional Ge-DG-JL TFET design and the numerical results associated to the conventional Si-DG-JL TFET [18]. it reveals that the proposed design with heterochannel outperforms considerably the conventional counterparts, with 58% improvement regarding the subthreshold swing factor and 54% enhancement in terms of the *I*_ON_/*I*_OFF_ ratio. The optimized design improves the device tunneling performance, not only through a more effective carrier-transport mechanism, but also through a distinctive reduction of the undesired ambipolar conduction.

**Table 1:** Overall electrical comparison of FoMs.

	conventional Ge-DG-JL TFET design	proposed Si_1−_*_x_*Ge*_x_*/Si/Ge DG-HJ-JL TFET structure	conventional Si-JL TFET structure [18]

design variables			

channel length *L*_g_ (nm)	20	20	20
source/drain extensions length *L*_s/d_ (nm)	10	10	25
dielectric permittivity	25	25	25
oxide thickness *t*_ox_ (nm)	2	2	2
channel thickness *t*_ch_ (nm)	5	5	5
gate work function (eV)	4.3	4.3	4.3
gated source work function (eV)	5.6	5.6	5.9
channel doping concentration *N*_d_ (cm^−3^)	1·10^19^, n-type	1·10^19^, n-type	1·10^19^, n-type
drain voltage *V*_ds_ (V)	1	1	1
Ge mole fraction of Si_1−_*_x_*Ge*_x_* source	—	60	—

performance parameters			

subthreshold swing (mV/dec)	114	42	81
*I*_ON_/*I*_OFF_ ratio (dB)	79	115	98
ambipolar conduction	high	suppressed	high

## Conclusion

In this work, a new DG-JL TFET design with a heterochannel (Si_1−_*_x_*Ge*_x_*/Si/Ge) has been proposed as a new way to achieve enhanced electrical performance and suppressed ambipolar conduction. It has been concluded from the obtained results that the investigated DG-HJ-JL TFET design offers the possibility to overcome the trade-off between improved switching characteristic and superior derived current capability. In addition, the impact of the Ge concentration on the electrical behavior of the device has been analyzed. It has been deduced that the proposed design with 60% of Ge provides an *I*_ON_/*I*_OFF_ ratio of 115 dB and a swing factor of 42 mV/dec. It has been also concluded that the optimized design offers superior scaling capability compared to the conventional Ge-DG-JL TFET structure. Therefore, the optimized design opens up the route for achieving an enhanced derived current capability with suppressed ambipolar conduction and for improving the device subthreshold behavior at the nanoscale level. This makes the optimized Si_1−_*_x_*Ge*_x_*/Si/Ge DG-HJ-JL TFET design a potential alternative for high-performance nanoelectronic applications. Moreover, this study can be extended by investigating the impact of other parameters such as the interfacial defects between both Si and SiGe materials and the degradation-related ageing effects including stress. To do so, new complex models and numerical simulations need to be developed.
